# The influence of latitude, geographic distance, and habitat discontinuities on genetic variation in a high latitude montane species

**DOI:** 10.1038/s41598-018-29982-7

**Published:** 2018-08-07

**Authors:** J. A. Hindley, B. A. Graham, P. C. Pulgarin-R., T. M. Burg

**Affiliations:** 1 Department of Biological Sciences, University of Lethbridge, 4401 University Dr. W, Lethbridge, AB T1K 3M4 Canada; 2 Laboratorio de Biología Evolutiva de Vertebrados, Departamento de Ciencias Biológicas, Universidad de Los Andes, Bogota, Colombia; 3 Facultad de Ciencias y Biotecnología, Universidad CES, Medellin, Colombia

## Abstract

Examining the factors that influence contemporary genetic patterns is important given the alarming rate at which natural environments are changing. In particular habitat fragmentation and climate change are expected to influence the distribution and diversity of natural populations. In this study we used both mitochondrial control region (mtDNA) and microsatellite data to answer the following questions about genetic diversity and divergence in mountain chickadees (*Poecile gambeli*) a resident bird species in western North America: (1) Do populations exhibit similar levels of genetic diversity across the range? (2) What is the genetic affinity of western populations in Oregon and Washington? (3) Do genetic patterns exhibit isolation by distance, or are genetic patterns more heavily influenced by habitat discontinuity? We tested the effects of isolation by distance and habitat distribution on genetic structure by analyzing 266 samples from 17 sites across western Canada and the United States. We found a near significant relationship between genetic diversity and latitude, however, our results indicate that overall, latitude is not a strong predictor of genetic diversity. Our analyses of populations in Oregon and Washington revealed a mismatch between patterns detected with mtDNA and microsatellite data. In particular, Washington clustered with the Coast Range/Cascades/Rocky Mountain mtDNA group, but with populations in southern Oregon/California based on microsatellite data. These results suggest the presence of a contact zone in Washington between the two mtDNA clades Coast Range/Cascades/Rocky Mountain and southern Oregon/California clades. Finally, our study revealed a greater effect of isolation by distance than isolation by habitat for both mtDNA and microsatellite data. Overall the isolation by distance signal was greater for mtDNA than microsatellite patterns. The greater signal of isolation by distance on mtDNA patterns likely reflects the strong effects of Pleistocene glaciations in shaping genetic patterns in western North America.

## Introduction

Although life history and historical processes are the primary factors shaping an organism’s evolutionary history, one must consider the role of contemporary processes on genetic variation^1–3^. Contemporary processes are especially important given the alarming rate that environments are changing and the effects of these changes on populations^4–6^. One only has to look at the Pleistocene glaciations to see the historical impact of climate change on population genetic structure. The fragmentation of populations due to repeated habitat contractions and expansions led to many recent speciation events^7^ and fragmented habitat including the separation of coniferous forests in western North America^8^. In the Pacific Northwest, the legacy of the last glacial maximum (LGM) combined with contemporary landscape features on population genetic structure is especially evident. Within this region, many subalpine species exhibit concordant genetic breaks associated with habitat fragmentation and isolation across arid, low elevation barriers, emphasizing the role of habitat discontinuity on genetic diversity and population differentiation^9–14^.

Environmental change (i.e. habitat fragmentation) impacts not only the landscape, but also behaviour and demographic factors. Species with strong year-round philopatry and infrequent dispersal will be heavily impacted, especially species with highly structured populations^15,16^. By comparison organisms with seasonal migration have higher dispersal potential and lower levels of population genetic structure^17,18^ (although see^19^). The higher genetic homogeneity may mean individuals are not as highly adapted to local conditions and may be able to survive in a variety of areas.

Birds are a good model group for examining the influence of landscape features on dispersal because of their exceptional dispersal capabilities and ability to move large distances^20^. Despite being capable of dispersing long distances, geographic and ecological barriers can impede dispersal between populations. Rivers, mountain ranges, and habitat discontinuities have all been shown to limit dispersal and promote genetic differentiation between populations^21–23^.

Given the presence of geographic and ecological barriers and the glacial history of the region, western North America is an ideal area to examine the effect of landscape and dispersal barriers on population genetic structure. Many sedentary species exhibit restricted dispersal across these barriers^14,21^ including the mountain chickadee (*Poecile gambeli*). Previous work on mountain chickadees demonstrated two well-supported mitochondrial DNA (mtDNA) groups, an eastern (Rocky Mountains and Great Basin) and a western (Sierra Nevada and Cascades) clade^24^, although a recent study suggested contemporary gene flow may be occurring between these two clades^25^. A common year-round resident of dry, montane, coniferous forests in western North America, the mountain chickadee is thought to have limited natal dispersal, exhibit strong philopatry, and limited altitudinal migration during winter^26^. Given that this species is thought to have limited dispersal capabilities, it is well suited for studies examining the effects of dispersal and biogeographic barriers on population genetic structure and phylogeographic structure^25,27,28^.

Here we examine phylogeographic and population genetic structure in mountain chickadees using the rapidly evolving mtDNA control region and microsatellite markers. In addition to exploring genetic structure, we sought to answer the following questions: (1) Do populations exhibit similar levels of genetic diversity across the range? Given that their range includes areas that were glaciated during the LGM, it allows us to test if younger populations (i.e. those populations in previously glaciated regions) exhibit similar levels of genetic diversity as older populations (i.e. those populations found in areas that were free of ice during the LGM); (2) What is the genetic affinity of western populations in Oregon and Washington? Previous research found eastern and western clades, but did not sample the area separating these two groups. The Pacific Northwest is a well-documented contact zone for many other species^29–31^; (3) Do genetic patterns exhibit isolation by distance, or are genetic patterns more heavily influenced by breaks in habitat? Mountain chickadees display strong philopatry and limited altitudinal migration, which suggests that distance between sites may reduce gene flow. By comparison mountain chickadees are primarily found in montane coniferous forests, and therefore habitat isolation and not geographic distance may influence genetic differentiation. Therefore, comparing these two variables, geographic distance and habitat isolation, will allow us to examine the roles of each variable on genetic differentiation in this species.

## Results

We observed high levels of genetic diversity in our analysis of mountain chickadee control region sequences and microsatellite markers (Table 1). For populations with ≥5 individuals, mean haplotype diversity ranged from 0.64 in south-central California to 0.93 in eastern Montana and northeast Oregon, while nucleotide diversity ranged from 0.001 (Colorado and south-central California) to 0.005 (Idaho). Within each population, the seven microsatellite loci showed variable levels of genetic diversity (Table 1); allelic richness across all populations ranged from 3.53 (Idaho) to 4.46 (south-central California), while observed heterozygosity ranged from 0.67 (southern California) to 0.93 (British Columbia-Revelstoke). Despite this range, we observed no significant differences in allelic richness or observed heterozygosity across populations (allelic richness: F_13,84_ = 1.20, p = 0.30) observed heterozygosity: F_13,84_ = 0.41, p = 0.96).

**Table 1:** Microsatellite and mitochondrial genetic diversity statistics within populations of mountain chickadee; latitude, longitude, number of samples screened at microsatellite loci (N_msat_), private alleles (*P*_*A*_), allelic richness (*A*_*R*_), observed heterozygosity (*H*_*o*_), expected heterozygosity (*H*_*e*_), number of samples sequenced for mtDNA control region (*N*_*mtDNA*_), number of haplotypes (*H*), nucleotide diversity (*π*) and haplotype diversity (*H*_*d*_). Allelic richness was only calculated for those populations with n > 5 individuals genotyped.

Population	Latitude	Longitude	N_msat_	P_A_	A_R_	H_o_	H_e_	N_mtDNA_	H	π	H_d_
Northwest British Columbia (NWBC)	58.51	−130.02	2	0	—	0.86	0.61	2	2	0.005	1.00
British Columbia Revelstoke (BCR)	51.04	−118.13	4	1	—	0.93	0.76	2	2	0.012	1.00
Central British Columbia (CBC)	54.75	−127.25	9	0	4.05	0.80	0.81	7	4	0.003	0.71
Southern Alberta (SAB)	49.35	−114.42	23	3	4.22	0.83	0.82	16	9	0.003	0.82
Western Montana (WMT)	46.54	−112.11	23	4	4.41	0.78	0.86	12	6	0.004	0.77
Eastern Montana (EMT)	46.66	−111.73	8	0	4.34	0.70	0.75	6	5	0.004	0.93
Colorado (CO)	39.77	−105.38	38	5	4.40	0.82	0.85	15	6	0.001	0.65
Utah (UT)	41.45	−111.50	20	2	4.28	0.81	0.83	17	11	0.003	0.85
Arizona (AZ)	35.15	−111.65	5	1	—	0.79	0.68	2	2	0.004	1.00
Washington (WA)	46.90	−121.64	20	0	4.06	0.76	0.81	18	8	0.003	0.74
Idaho (ID)	46.84	−116.96	10	2	3.53	0.76	0.80	5	5	0.005	0.90
Northeast Oregon (NEOR)	44.96	−118.23	25	3	4.28	0.78	0.83	19	12	0.003	0.93
Central Oregon (CEOR)	44.43	−120.92	17	1	4.21	0.82	0.83	15	4	0.002	0.70
Southern Oregon (SOR)	42.70	−122.15	24	1	4.05	0.77	0.82	19	9	0.002	0.82
Central California (CCA)	40.31	−123.10	12	4	3.86	0.80	0.79	12	8	0.003	0.85
South-central California (SCCA)	35.72	−118.15	11	2	4.46	0.74	0.80	8	3	0.001	0.64
Southern California (SCA)	34.16	−116.80	15	2	3.89	0.67	0.79	15	6	0.002	0.71

**Figure 1:**
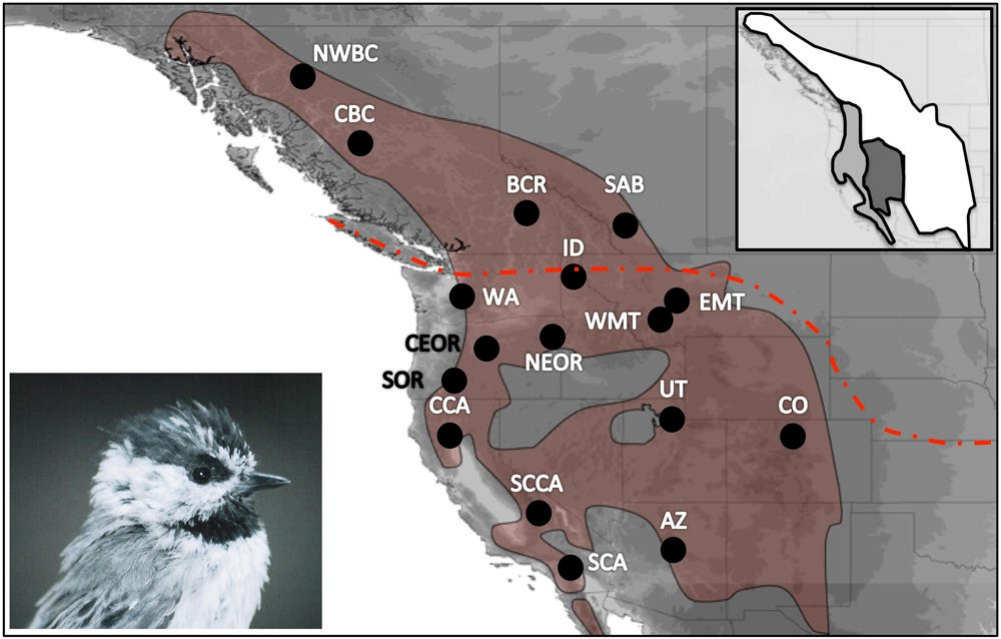
Range map showing the sampling sites for mountain chickadees (lower inset; picture taken by Brendan Graham) in western North America. The red dashed line indicates the extent of glaciation during the Last Glacial Maximum. Top right inset shows three major morphological groups previously described in Behle^69^: *gambeli –*white, *inyoensis* – dark grey, *baileyae –* light grey. Sampling sites include central British Columbia (CBC), northwest BC (NWBC), Revelstoke, BC (BCR), southern Alberta (SAB), western Montana (WMT), eastern Montana (EMT), Colorado (CO), Utah (UT), Arizona (AZ), Washington (WA), Idaho (ID), northeast Oregon (NEOR), central OR (CeOR), southern Oregon (SOR), central California (CCA), south central CA (SCCA), and southern CA (SCA). Range maps were created in DIVA-GIS 7.5 (www.diva-gis.org) using digital distribution files provided by Ridgley *et al*.^70^.

Our analysis of genetic diversity along a north-south transect (Fig. 1) revealed a near-significant, positive relationship between latitude and mtDNA genetic diversity (adjusted r^2^ = 0.15, t = 1.83, p = 0.09). By comparison, we observed a negative non-significant relationship between latitude and microsatellite genetic diversity (adjusted r^2^ = −0.08, t = −0.01, p = 0.99). For our RDA, latitude was a near-significant predictor of mtDNA genetic diversity (F_1,12_ = 3.36, p = 0.10), and accounted for 21.86% of the observed variation. Similarly latitude was a near significant predictor of microsatellite genetic diversity (F_1,12_ = 0.01, p = 0.99), and accounted for <1% of the observed variation.

### Phylogeographic and population structure

**Figure 2:**
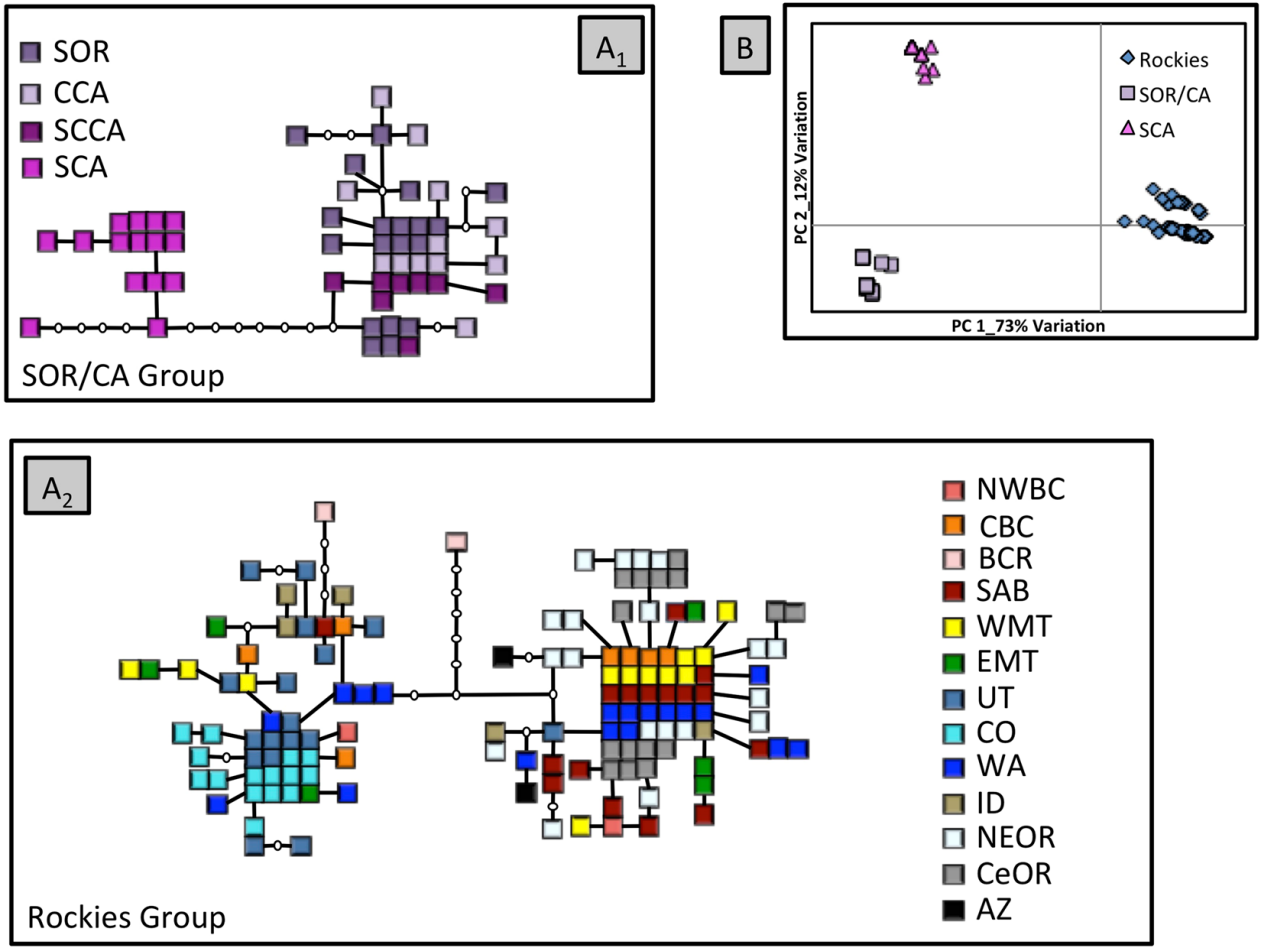
(**A**) Statistical parsimony network of mtDNA haplotypes for mountain chickadees: A_1_ SOR/CA group; A_2_ Rockies group. Each square represents a single individual and open circles indicate inferred haplotypes. Refer to Fig. 1 for location of sampling sites. (**B**) Principal coordinate analysis of mtDNA data based on population location. Coordinate 1 explains 73% of the variation and coordinate 2 12%.

**Figure 3:**
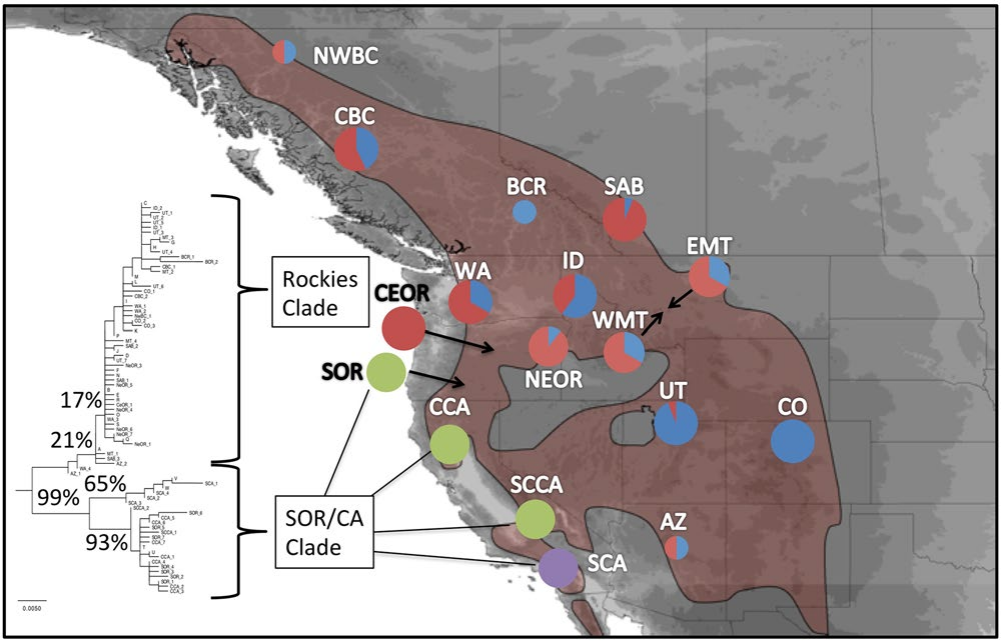
Unrooted ML tree with bootstrap values (left) and BAPS 95% CI cluster assignment (K = 4) of mtDNA. Contemporary mountain chickadee distribution is outlined in main map, populations with small circles representing sites where <5 individuals were sequenced, and larger circles where ≥5 individuals were sequenced. Colours represent the proportion of individuals that were assigned to each clade based on BAPS analysis. Green and purple circles represent individuals from the western clade (as outlined in Fig. 1), while blue and red represent haplotypes from the eastern clade. Range maps were created in DIVA-GIS 7.5 (www.diva-gis.org) using digital distribution files provided by Ridgley *et al*.^70^.

We identified a total of 80 control region haplotypes. Our statistical parsimony network revealed three distinct groups: (1) Coast Range/Cascades/Rocky Mountain; (2) southern Oregon/central California; and (3) southern California (Fig. 2A). Although we observed a number of shared haplotypes within each group (19 shared haplotypes were identified in Coast Range/Cascades/Rocky Mountains, and four in southern Oregon/central California), we did not observe any shared haplotypes among groups. The southern California group was separated by 16 fixed differences from the Coast Range/Cascades/Rocky Mountain group and by two fixed differences from the southern Oregon/central California group whereas the southern Oregon/central California group was separated by 18 fixed differences from the Coast Range/Cascades/Rocky Mountain group. Although our statistical parsimony network suggested three groups, our maximum likelihood tree only supported two distinct clades (Fig. 3). In contrast to the results of Spellman *et al*.^24^, northern populations in the Cascades and Coast Range (Washington and central Oregon) did not group with southern Oregon and California populations. Instead these populations grouped with populations in the Great Basin, Rocky Mountains and Canada.

Pairwise Φ_ST_ and F_ST_ values revealed significant differentiation in mountain chickadees (Table 2); 71 of 91 pairwise Φ_ST_ comparisons and 53 of 91 pairwise F_ST_ comparisons were significant following corrections for multiple pairwise tests. Southern California was significantly different from all populations based on Φ_ST_ and all populations with the exception of south-central California and eastern Montana based on F_ST_. Pairwise Φ_ST_ comparisons revealed that south-central California, central California, and southern Oregon were not significantly different from each other, but were significantly different from all Rocky Mountain populations. Unlike pairwise F_ST_ comparisons, where central California was significantly different from all populations, southern Oregon and south-central California were significantly different from only a handful of populations. Within the Rockies group 28 of the 45 pairwise Φ_ST_ comparisons were significantly different from each other, with four populations (central Oregon, northeast Oregon, Colorado and Utah) accounting for all but one of the significant Φ_ST_ values. Among Rocky Mountain populations 24 of 45 F_ST_ comparisons were significant, Idaho was significantly different from all populations, and northeast Oregon from all populations except southern Alberta and Utah, Central Oregon from all populations except southern Alberta, eastern and western Montana, and Colorado, and central British Columbia from all but three populations (southern Alberta, western Montana and eastern Montana).

**Table 2:**
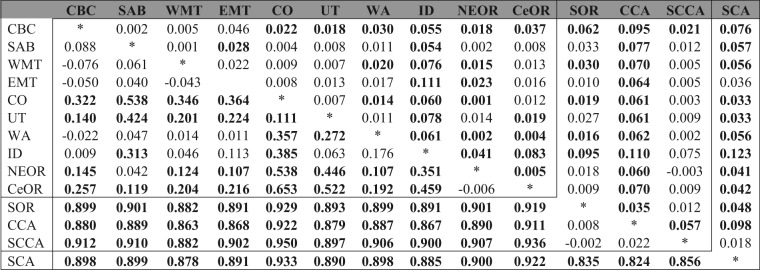
MtDNA Φ_ST_ (bottom left) and microsatellite F_ST_ (upper right) values for pairwise comparisons among mountain chickadee populations (bold = significant after Benjamini-Hochberg correction). AZ, NWBC, BCR populations were excluded due to small sample size (n < 8).

Similar to our statistical parsimony network, PCoA (Fig. 2B) showed three genetic clusters: Coast Range/Cascades/Rocky Mountain, southern Oregon/central California, and southern California. SAMOVA also grouped populations into the same three distinct clusters (F_CT_ = 0.85; p < 0.0001). The majority of variation was explained by differences among groups (85.43%; p < 0.001), although there was significant variation among populations within groups (3.86%; F_SC_ = 0.27; p < 0.001) and within populations (10.70%; F_ST_ = 0.89, p < 0.0001). In contrast to these previous methods, BAPS identified four distinct clusters; it split the Coast Range/Cascades/Rocky Mountains into two different groups. Every population with exception of central Oregon and Colorado had at least one individual with a haplotype from each of the two Coast Range/Cascades/Rocky Mountains groups identified with this analysis.

**Figure 4:**
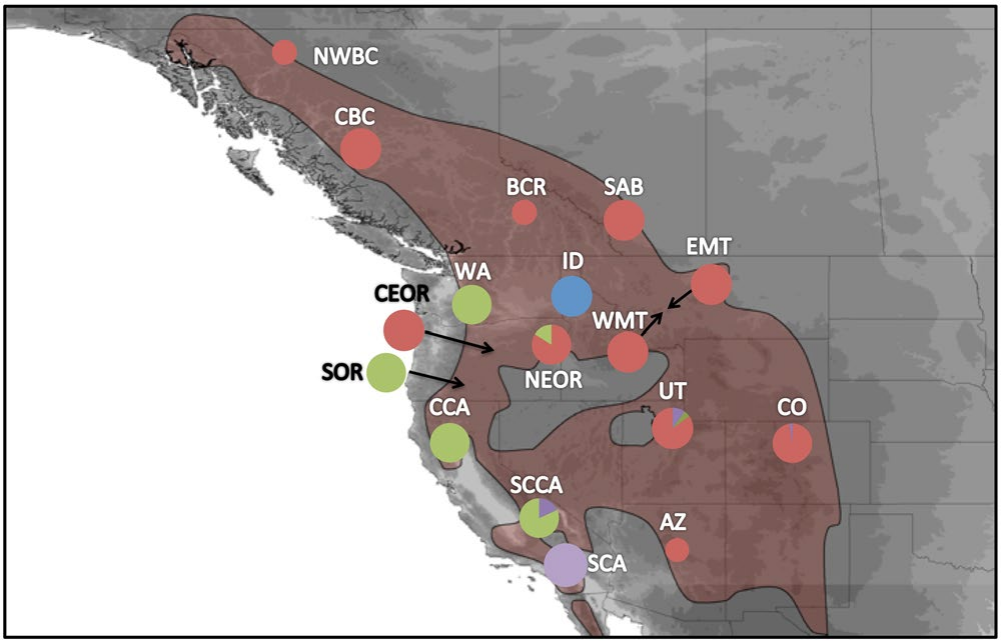
Proportion of individuals in each mountain chickadee population assigned to one of the four clusters by Structure based on microsatellite data. Individual birds were assigned to the cluster (each cluster indicated by different colour) with the highest *Q* value (ancestry coefficient). Populations with small circles represent sites where <8 individuals were genotyped, and larger circles represent sites where ≥8 individuals were screened. Range maps were created in DIVA-GIS 7.5 (www.diva-gis.org) using digital distribution files provided by Ridgley *et al*.^70^.

Structure revealed four distinct clusters (ΔK = 1.94; Pr Ln (X|K) = −7338.60; Fig. 4). Similar to mtDNA patterns, the majority of Rocky Mountain populations clustered together with central Oregon, although Idaho formed a separate cluster independent of all other Rocky Mountain populations. One other difference between mtDNA and microsatellite patterns, was that Washington clustered with southern Oregon, central California, and south-central California, whereas it clustered with Rocky Mountain populations based on mtDNA control region sequences. Similar to mtDNA analyses, southern California was distinct from all other populations. We observed some introgression between groups based on microsatellites with 11 of 264 individuals (4.2%) assigned to a cluster outside of their ‘home’ cluster (Fig. 4). The average Q of birds that assigned to their home cluster was 0.69, while those birds that showed evidence of introgression and assigned to an alternate cluster had an average Q of 0.47.

### Isolation by distance and resistance analyses

Comparisons of microsatellite and mtDNA patterns of differentiation revealed contrasting patterns. Whereas four explanatory variables (geographic distance, habitat resistance, latitude, and longitude) explained relatively little of the observed differentiation (0.5–1.2%; Table 3) for microsatellites, these four variables were better predictors of mtDNA differentiation (7.2–52.9%). When we controlled for the effects of geographic distance and habitat resistance, our conditional models revealed that geographic distance is a stronger predictor of mtDNA genetic differentiation than habitat resistance; geographic distance explained a greater proportion of the variance (46.8%) than habitat resistance (7.2%). By comparison geographic distance and habitat resistance explained a similar proportion of the variance for microsatellites (1.2% and 0.5% respectively) indicating that both of these variables are not strong predictors of microsatellite genetic differentiation patterns.

**Table 3:** Redundancy analysis models examining the effect of geographic distance, habitat resistance, latitude, and longitude on microsatellite and mtDNA genetic differentiation. Conditional tests were conducted to test the effect of geographic distance on genetic variation while controlling for habitat resistance along with the reciprocal test. Total variance (Inertia), percent of the variation explained (% Variation), degrees of freedom (df), F-Ratio (F), and p-values (p) are presented for each model.

Variable	Inertia	%Variation	df	F	p
Microsatellite					
Geographic Distance	2.16	1.2%	1,264	5.20	<0.001
Habitat Resistance	0.89	0.5%	1,264	2.12	<0.001
Latitude	2.04	1.2%	1,264	4.91	<0.001
Longitude	1.06	0.6%	1,264	2.52	0.003
Geographic Distance | Habitat Resistance	2.16	1.2%	1,263	1.99	0.020
Habitat Resistance | Geographic Distance	0.82	0.5%	1,263	5.07	<0.001
Mitochondrial DNA					
Geographic Distance	49.12	52.9%	1,188	265.09	<0.001
Habitat Resistance	6.66	7.2%	1,188	16.21	<0.001
Latitude	25.04	26.9%	1,188	79.90	<0.001
Longitude	10.38	11.2%	1,188	26.53	<0.001
Geographic Distance | Habitat Resistance	45.53	46.8%	1,187	241.08	<0.001
Habitat Resistance | Geographic Distance	1.07	1.2%	1,187	5.94	<0.001

## Discussion

In this study we sought to answer the following questions: (1) Do populations exhibit similar levels of genetic diversity across the range? (2) What is the genetic affinity of western populations in Oregon and Washington? (3) Do genetic patterns exhibit isolation by distance, or are genetic patterns more heavily influenced by breaks in habitat? Across the range, we found a near-significant positive correlation between latitude and mtDNA genetic diversity using both a linear model and RDA approach, and microsatellite genetic diversity using an RDA approach only. Although these results indicate that latitude may predict genetic diversity, latitude may be reflective of other processes including isolation and post-glacial colonization patterns for this species. Our inclusion of populations from Washington and Oregon provided critical insights into genetic patterns; in particular our results revealed a contact zone in Washington between southern Oregon/California and Coast Range/Cascades/Rocky Mountain mtDNA clades given that Washington grouped with southern Oregon/central California populations based on microsatellite patterns. This area has been reported as an area of contact in several other avian species^29,32^. Finally, we found that isolation by distance has had a strong effect on the genetic structure of mountain chickadees; specifically on mtDNA patterns Although habitat resistance explained less of the genetic variation in this species, we cannot ignore the important influence of habitat fragmentation as a result of Pleistocene glaciations similar to other studies^25,26^.

Our analyses of genetic diversity across the range suggest the potential for a correlation between genetic diversity and latitude. In particular mtDNA genetic diversity showed a stronger correlation with latitude than microsatellite genetic diversity, indicating that genetic diversity was higher in previously glaciated areas than ice-free areas. It is important to note though that this relationship may reflect both past and present processes, including isolation and post-glacial colonization patterns. For example work on post-glacial patterns of genetic diversity in Italian agile frogs (*Rana latastei*) found that the distance from glacial refugium and isolation were strong predictors of genetic diversity in this species^27^. In our own study, isolation may drive patterns of genetic diversity, given that our most isolated population in southern California exhibited some of the lowest levels of genetic diversity for both mtDNA and microsatellite markers. Additionally Idaho, a distinct population based on microsatellite markers also exhibits lower microsatellite genetic diversity and appears to be isolated from other populations in the Coast Range/Cascades/Rocky Mountain mtDNA group (see below). Further our analyses of population structure indicate limited gene flow is occurring between populations in the Rocky Mountains and central Oregon, and along the Pacific Coast among Washington, southern Oregon and California populations.

Previous genetic studies of mountain chickadees did not include or sampled relatively few individuals from Oregon and Washington^25,26^. Our inclusion of these populations allowed us to analyze the genetic affinity of these populations in an important biogeographic area with a complex geographic and climate history^28^. Individuals from Washington clustered with southern Oregon and California populations based on microsatellites and with Coast Range/Cascades/Rocky Mountain populations based on mtDNA. These contrasting patterns indicate a previously undescribed contact zone between western and eastern populations. Several other taxa show concordant genetic breaks in this area presumably following common vicariance events^28–31^. Outside of Washington, we found little evidence to suggest contemporary gene flow between eastern and western populations, in contrast to Manthey *et al*.^25^. Differences in genetic patterns between the two studies may reflect differences in the evolutionary history, as well as the resolution of the markers. Despite the subtle differences in genetic patterns, the overall pattern of Washington, southern Oregon, and California being genetically distinct from Coast Range/Cascades/Rocky Mountain populations was apparent in both studies.

Our combined analyses indicate geographic distance has a greater influence on genetic differentiation than habitat discontinuities, although both of these factors explained a relatively low portion of variation for microsatellite loci. This result is surprising as habitat discontinuities correspond to genetic differentiation in two closely related species with similar dispersal potential: chestnut-backed chickadees (*Poecile rufescens*)^32^ and black-capped chickadees (*Poecile atricapillus*)^23^. Perhaps contemporary mountain chickadee habitat is not as isolated as that of these two sister species, however, this seems unlikely. Mountain chickadees are specialists inhabiting dry coniferous forests at high elevation, so their distribution range may be more disjunct than it appears. Therefore we must also consider that although our sampling is adequate enough to detect isolation by distance, it may be too coarse of a scale to detect isolation by habitat resistance. Thus a more appropriate sampling scale at a regional scale may be required to detect a pattern of isolation by habitat resistance.

Habitat resistance was a strong predictor of variation for mtDNA; this pattern likely reflects the glacial history of western North America^8^. Large portions of the mountain chickadees present distribution were covered by ice sheets and alpine glaciers resulting in habitat fragmentation and restricted movement between eastern and western groups^26,33^. Further, large, arid, treeless basins are present within the range of this species, separating populations in southern and south-central California from central California. These arid, treeless basins restrict dispersal, as evident by a north/south break in that area in a variety of species^34–37^. Finally we cannot rule out the possibility that geographic distance may be masking the effects of habitat fragmentation. Mountain chickadees exhibit strong philopatry and limited altitudinal migration and these behaviours may amplify the effect of isolation by distance in our study.

MtDNA patterns indicate historical isolation of mountain chickadee populations and continued isolation of maternal lineages, as we found no shared haplotypes between western and eastern populations. Further, we found clear north/south divisions within the western clade, as indicated by pairwise Φ_ST_ comparisons, a pattern that was undetected or incomplete in previous mtDNA studies of mountain chickadees^25,38^. Differences between studies partially reflect differences in markers and sampling. Although ND2 and control region have the same genealogical histories^14^, the control region exhibited greater resolution (as has been reported in other studies^39^) in our study than the ND2 marker used by Spellman *et al*.^24^.

In addition to genetic patterns indicating historical isolation, our results also suggest evidence for recent isolation in mountain chickadees. For example, a fourth genetically distinct group was detected with microsatellite data in Idaho. Idaho was completely distinct from other populations based on Structure and pairwise F_ST_ comparisons despite being with all Coast Range/Cascades/Rocky Mountain populations based on mtDNA data. Adams and Burg^23^ found that black-capped chickadee populations in Idaho were genetically distinct from northern population in British Columbia and Alberta, as well as populations in Montana and southern Alberta. They attributed this pattern to reduced connectivity between forested areas as a result of mountain ranges and large canyons, restricting gene flow between populations. Therefore biogeographic features in this region may explain the isolation of this population.

The reduced genetic differentiation between Colorado, Utah, northeast Oregon, and central Oregon may be indicative of increased contemporary gene flow in mountain chickadees as suggested by Manthey *et al*.^25^. Sex-biased dispersal may explain differences between mtDNA and microsatellites^40^, although female-biased dispersal is more common in avian species^20^.

The mountain chickadee exhibits strong phylogeographic and population genetic structure. Our inclusion of Pacific Northwest populations revealed a contact zone between southern Oregon and California populations and Coast Range/Cascades/Rocky Mountain populations based on contrasting patterns for mtDNA and microsatellite data. MtDNA and microsatellite patterns were primarily concordant, indicating that both historical and contemporary gene flow are restricted between populations. Although isolation by distance exhibited a greater effect than habitat resistance in our analyses, we cannot discount the effect of habitat on genetic patterns. Overall our study indicates the important effects that Pleistocene glaciations have had on genetic patterns, especially in resident species like the mountain chickadee. Further our study highlights the complex biogeographic history of northwestern North America where the interaction between historical process, barriers to dispersal, and habitat configuration have influenced genetic diversity and divergence^28^. Further work is necessary to determine the variables that shape contemporary genetic patterns within this diverse region.

## Methods

### Sampling

A total of 266 blood and tissue samples from 17 sampling sites were collected across the contemporary mountain chickadee range (Fig. 1; Table 1). Birds were captured using mist nets, and blood and/or feather samples were collected from 200 individuals during the summer of 2008 to 2010 and stored in ethanol (95%). All samples at a single sampling site (hereafter referred to as a population) were collected within a 50 km radius. Sixty-six samples collected within the last 20 years were obtained from museums (see acknowledgements). DNA was extracted from whole blood or tissue using a modified chelex method^38,40^. All protocols were approved by the University of Lethbridge animal care board, and all methods in this study were performed in accordance with the relevant guidelines and regulations.

### MtDNA amplification and sequencing

Two PCR primers, H1015 (5′-CGCGGGTTTAACGAATGTGG-3′) and LmochCR1 (5′-CAGGGTATGTATGTCTTTGCATTC-3′; designed this study), were used to amplify a 765 bp product within Domains I and II of the control region for 190 samples. The polymerase chain reaction (PCR) was carried out in an Eppendorf Mastercycler. PCR consisted of approximately 100 ng of template DNA, 1 μM of each primer, 200 μM dNTP, 2.5 mM MgCl_2_, 1 unit of Taq DNA polymerase (Crimson) and PCR buffer (Crimson or Promega) in a final volume of 25 μl. Amplification consisted of one cycle at 95 °C for 2 min, 54 °C for 45 s, and 72 °C for 60 s; 37 cycles of 94 °C for 30 s, 54 °C for 45 s, and 72 °C for 60 s; and one final cycle at 72 °C for 5 min. The PCR products were sequenced using an Applied Biosystems 3130 Genetic Analyzer or sent to Genome Quebec for sequencing (McGill University, Montreal, QC, Canada). MtDNA chromatograms were checked and sequences manually aligned in MEGA v5.0^39^.

### Microsatellite genotyping

Seven microsatellite primer pairs isolated from other passerine species (Escu4, Escu6, Pat14, Pdo5, Ppi2, Titgata02 and Titgata39) were used for genotyping^41–45^. All forward primers were modified with the addition of M13 sequence to the 5′ end to allow for direct incorporation of a fluorescently labeled M13 primer. PCR reactions consisted of approximately 100 ng of template DNA, 0.5 μM of each microsatellite primer and 0.05 μM M13 tag, 200 μM dNTP, 1–2 mM MgCl_2_ (see below), 0.5 units of Crimson Taq DNA polymerase (New England BioLabs) and PCR buffer in a final volume of 10 μl. MgCl_2_ concentration varied depending on the locus (2 mM for Escu4, Titgata02, Titgata39 and Pat14, 1.5 mM for Escu6 and Ppi2, and 1 mM for Pdo5) and 1% formamide was added to the PCR for Escu4 and Ppi2. All loci were amplified using a two-step annealing procedure: one cycle for 2 min at 94 °C, and 45 s at T_A1_, 1 min at 72 °C; 7 cycles of 1 min at 94 °C, 30 s at T_A1_, 45 s at 72 °C; 31 cycles of 30 s at 94 °C, 30 s at T_A2_, 45 s at 72 °C; and one final extension of 5 min at 72 °C. For loci Escu4 and Pdo5 T_A1_ = 45 °C and T_A2_ = 48 °C, and for the other five loci T_A1_ = 50 °C and T_A2_ = 52 °C. The PCR was carried out in an Eppendorf Mastercycler and PCR products were run on a 6% acrylamide gel using a Li-COR 4300 (Li-COR Inc.) with appropriate controls and size standards. All microsatellite genotypes were visually scored independently by two people (JAH and TMB). Finally we re-amplified and ran a subset of individuals to ensure that we accurately scoring across gels.

### Genetic diversity analyses

To measure genetic diversity for mtDNA, we calculated the number of haplotypes (H), haplotype diversity (*H*_*d*_), and nucleotide diversity (π) using DnaSP v5.10^46^.

We tested all microsatellite loci x population combinations for deviations from Hardy-Weinberg equilibrium (HWE) and linkage disequilibrium (LD) using GENEPOP 4.0.10^47,48^. We found no significant linkage disequilibrium between loci (p > 0.77), but following corrections for multiple tests six of 119 population x locus comparisons deviated from HWE: western Montana at locus Pdo5 (p < 0.001), southern Oregon at Ppi2 (p < 0.001), Washington at Titgata02 (p = 0.030), south-central California at Pat14 (p = 0.003) and southern California for Pdo5 (p < 0.001) and Titgata39 (p = 0.017). As no locus or population showed consistent deviations from HWE, we used all seven loci for subsequent analyses. We calculated observed heterozygosity, expected heterozygosity, and allelic richness for each population using Fstat 1.2^49^. Finally, we compared microsatellite genetic diversity between populations using an Analysis of Variance in PAST version 3^50^.

Rather than using single genetic metric (e.g. observed heterozygosity) to arbitrarily measure genetic diversity^51^, we followed the methodology of Ficetola *et al*.^27^ and performed a principal component analysis (PCA) to estimate genetic diversity at each population for both our mtDNA and microsatellite datasets. For our mtDNA dataset we included nucleotide diversity and haplotype diversity in our PCA; the first principal component (Eigen value = 1.69) explained 84.43%. For our microsatellite dataset, we included allelic richness, observed heterozygosity, and the number of private alleles; again we retained only a single principal component (Eigen value = 1.47), which explained 48.88% of the variance.

To compare patterns of genetic variation between new populations (populations in previously glaciated areas) and old populations (areas that were ice free during the LGM; refer to Fig. 1), we plotted genetic diversity against latitude. For this analysis we constructed two separate models, using the first principal component for summarizing mtDNA genetic diversity and the first principal component summarizing microsatellite genetic diversity as our response variables, and latitude as our fixed variable; we analyzed the phylogeographic relationship between genetic diversity and latitude using linear regression models with the lme4 package in R^52^. For these analyses we only included populations with more than eight samples and therefore we excluded northwest British Columbia, British Columbia Revelstoke, and Arizona.

To further examine the relationship between genetic diversity and latitude, we performed a Redundancy analysis models^53^. RDA extends on multivariate linear regression approaches by examining the effects of explanatory variables on a given response variable. As opposed to providing a correlation coefficient, RDA follows an ANOVA approach and provides F-ratios and variance explained by each explanatory variable, thereby allowing a more nuanced interpretation of results^54^. For these analyses, we used the first principal component summarizing mtDNA genetic diversity and the first principal component summarizing microsatellite genetic diversity as our response variables and latitude as our explanatory variable. We performed this analysis using the Vegan package in R.

### Phylogeographic and population structure

Two different phylogenetic approaches, statistical parsimony and maximum likelihood, were used to determine the phylogeographic relationship among the 190 chickadee mtDNA sequences. A statistical parsimony network was constructed using the program TCS^55^ with gaps treated as a fifth character state. The program jModeltest^56^ was used to select the model of sequence evolution that best fit the sequence data (HKY + G + I; AIC = 3959.94), and a maximum likelihood (ML) tree was constructed in MEGA using the same substitution model (discrete gamma categories n = 4) and nearest neighbor interchange heuristic model, with 1000 bootstrap replicates to evaluate robustness.

We calculated pairwise Φ_ST_ (mtDNA) and F_ST_ (microsatellite) values for all populations with at least eight samples (Table 1). Pairwise Φ_ST_ values were calculated in Arlequin v3.0 (10 000 permutations)^57^, while pairwise F_ST_ was calculated from microsatellite data using GENODIVE v2.0b20 (10, 000 permutations)^58^. All p-values were corrected for multiple tests using the Benjamini-Hochberg false discovery rate (FDR) correction^59,60^. We used FDR corrections, given that previous studies have indicated that Bonferroni corrections often result in an increase of Type II errors, where the number of significant pairwise comparisons is underestimated^60^.

We used three different approaches to examine population structure based on our mtDNA dataset. First we used a Principal Coordinate Analysis (PCoA) to examine if haplotypes clustered geographically. For this analysis we created a distance matrix using our pairwise Φ_ST_ values and then performed a PCoA using GenAlEx 6.5 ^61^. Next we used the Bayesian clustering program BAPS 5.3^62^ to estimate the number of clusters. We conducted 10 runs with the maximum number of possible clusters set at 17; we did not use sampling location as a prior. Individuals were assigned to the cluster with the highest average *Q* value (ancestry coefficient) from the 10 runs at the optimal K. Finally, we conducted a spatial analysis of molecular variance using SAMOVA^63^ to identify groups of sampling sites that are geographically homogenous and maximally differentiated from each other. SAMOVA uses a simulated process to define groups maximizing the proportion of total genetic variance due to differences between groups and, unlike AMOVA and SAMOVA, does not require groups to be defined *a priori*.

We examined genetic differentiation for microsatellites using the Bayesian clustering program Structure v2.3^64^. To estimate the most likely number of clusters, we ran each K (1–10) for 10 iterations, using a burn-in of 50,000 and a run of 100,000 steps^65^. We ran this analysis for all 17 populations using the admixture model, correlated alleles, and sampling location as priors. We estimated K by calculating ΔK^65^ in Structure Harvester^66^ and examining posterior probabilities as suggested by Pritchard *et al*.^64^. Following our initial STRUCTURE analyses, we explored the potential for hierarchical structure by performing subsequent runs on each of the identified clusters. We found no evidence of further population substructure in any of the subsequent runs.

### Habitat Resistance Analysis

To get a measure of habitat connectivity and resistance between sampled individuals of mountain chickadee, we used graph theory to calculate distance and routes as implemented in the package gdistance in R^67^. It uses a raster (cell values) that represent a property of the landscape, in this case, tree-habitat. We created a least cost resistance matrix using latitude and longitude of all sampled mountain chickadees, and we used a global map of tree diversity^68^ to extract habitat tree density (habitat) values for each coordinate point. The global map of tree diversity was restricted to the distribution of the mountain chickadee in the USA and Canada. The resistance values calculated for each pair of individuals was used to explain genetic variation for both microsatellites and mtDNA. All geographic analyses were done in R statistical program.

### Isolation by distance and habitat resistance analyses

Similar to our analyses of genetic diversity, we used Redundancy analysis Models to examine the effect of distance and habitat resistance on genetic differentiation^53^. For these analyses, we specifically used distance based-Redundancy Analyses (dbRDA) to examine the effects of geographic distance, habitat resistance, latitude, and longitude on individual genetic variation for both microsatellite and mtDNA data. We analyzed each dataset individually and used the *capscale* function in the R-package VEGAN to perform all analyses^52^. We calculated the Cavalli-Sforza chord distance in GENODIVE for our microsatellite data and for our mtDNA data set we calculated Nei’s genetic distance using GenAlEx for our response variables. For our explanatory variables we used the decimal degree geographic coordinates (longitude and latitude) of each individual to test the effect of geographic distance. Next, we reduced the habitat distance matrix (as described above) to a single continuous variable using a Principal Coordinate Analysis in GenAlEx, retaining the first significant Principal Coordinate which explained 57% of the variation; p < 0.05 as our explanatory variable. We tested the effect of latitude and longitude separately to determine if north-south or east-west differences influenced genetic variation. In addition to testing the explanatory variables separately, we performed conditional tests where we controlled for the effect of geographic distance on habitat resistance, as well as the reciprocal test to determine which factors primarily influence genetic variation.

All microsatellite genotyping will be archived in Dryad and all mtDNA sequences will be archived in GenBank.
