# Author Correction: The influence of latitude, geographic distance, and habitat discontinuities on genetic variation in a high latitude montane species

**DOI:** 10.1038/s41598-018-36713-5

**Published:** 2018-12-06

**Authors:** J. A. Hindley, B. A. Graham, P. C. Pulgarin-R., T. M. Burg

**Affiliations:** 1 Department of Biological Sciences, University of Lethbridge, 4401 University Dr. W, Lethbridge, AB T1K 3M4 Canada; 2 Laboratorio de Biología Evolutiva de Vertebrados, Departamento de Ciencias Biológicas, Universidad de Los Andes, Bogota, Colombia; 3 Facultad de Ciencias y Biotecnología, Universidad CES, Medellin, Colombia

Correction to: *Scientific Reports* 10.1038/s41598-018-29982-7, published online 07 August 2018

In this Article, the Data availability section was omitted from the Additional Information section:

## Data Availability

All sequences used for the mtDNA analyses in this project have been deposited in genbank accession numbers MK055563 - MK055752.

